# Reducing the Oxidation Level of Dextran Aldehyde in a Chitosan/Dextran-Based Surgical Hydrogel Increases Biocompatibility and Decreases Antimicrobial Efficacy

**DOI:** 10.3390/ijms160613798

**Published:** 2015-06-16

**Authors:** Maggie Chan, Heather J. L. Brooks, Stephen C. Moratti, Lyall R. Hanton, Jaydee D. Cabral

**Affiliations:** 1 Department of Microbiology & Immunology, University of Otago, 9054 Dunedin, New Zealand; E-Mails: m.chan@massey.ac.nz (M.C.); heather.brooks@otago.ac.nz (H.J.L.B.); 2 Department of Chemistry, University of Otago, 9054 Dunedin, New Zealand; E-Mails: smoratti@chemistry.otago.ac.nz (S.C.M.); lhanton@chemistry.otago.ac.nz (L.R.H.)

**Keywords:** hydrogel, biocompatibility, chitosan, oxidized dextran, antimicrobial

## Abstract

A highly oxidized form of a chitosan/dextran-based hydrogel (CD-100) containing 80% oxidized dextran aldehyde (DA-100) was developed as a post-operative aid, and found to significantly prevent adhesion formation in endoscopic sinus surgery (ESS). However, the CD-100 hydrogel showed moderate *in vitro* cytotoxicity to mammalian cell lines, with the DA-100 found to be the cytotoxic component. In order to extend the use of the hydrogel to abdominal surgeries, reformulation using a lower oxidized DA (DA-25) was pursued. The aim of the present study was to compare the antimicrobial efficacy, *in vitro* biocompatibility and wound healing capacity of the highly oxidized CD-100 hydrogel with the CD-25 hydrogel. Antimicrobial studies were performed against a range of clinically relevant abdominal microorganisms using the micro-broth dilution method. Biocompatibility testing using human dermal fibroblasts was assessed via a tetrazolium reduction assay (MTT) and a wound healing model. In contrast to the original DA-100 formulation, DA-25 was found to be non-cytotoxic, and showed no overall impairment of cell migration, with wound closure occurring at 72 h. However, the lower oxidation level negatively affected the antimicrobial efficacy of the hydrogel (CD-25). Although the CD-25 hydrogel’s antimicrobial efficacy and anti-fibroblast activity is decreased when compared to the original CD-100 hydrogel formulation, previous *in vivo* studies show that the CD-25 hydrogel remains an effective, biocompatible barrier agent in the prevention of postoperative adhesions.

## 1. Introduction

Bioabsorbable hydrogels as effective post-operative aids continue to gain much interest due to their significant water content, adjustable mechanical properties, flexibility, and ability to mimic living tissue [1]. Naturally derived polysaccharides, such as chitosan and dextran, are of particular interest due to their biocompatibility, biodegradability, ease of application, and the ability to form gels *in situ* [2]. Examples of naturally-derived polysaccharides used as mechanical barriers in current therapies include Seprafilm (Genzyme) [3,4] and Interceed (Ethicon) [5,6]. Following surgical procedures, these therapies are used to separate internal organs and tissues so that they do not adhere to each other during the healing process. Our group has developed a chitosan/dextran-based (CD) hydrogel as a post-surgical adjunct for use in endoscopic sinus surgeries (ESS). The CD hydrogel is formed by cross-linking between succinylated chitosan (SC) and dextran aldehyde (DA).

Chitosan is the second most abundant polysaccharide in nature after cellulose. It was selected as a hydrogel component due to its well characterized biocompatibility, biodegradability, hemostatic, and antimicrobial activities. It is widely used for drug delivery, as a food additive, and in tissue engineering [7]. Chitosan, derived from the alkaline deacetylation of chitin, is a linear polysaccharide consisting of (1, 4)-linked 2-amino-deoxy-β-d-glucan. Depending on its application, the chemical characteristics of chitosan can be altered by modifying the degree of acetylation and its molecular weight [8]. In order to make chitosan soluble at neutral pH and in aqueous conditions, SC was synthesized by the introduction of succinyl groups on the N-terminus of chitosan’s glucosamine units using succinic anhydride. However, SC does not possess chitosan’s antibacterial activity due to the loss of chitosan’s polycationic structure, as SC is negatively charged [9].

Dextran, the other hydrogel component, was selected due its biocompatibility, solubility in polar solvents, and ease of chemical modification, making it an attractive material for designing functional polymers. Dextran is composed of a linear backbone of α-linked d-glucopyranosyl repeating units with different proportions of linkages and branches. The branched bonds are represented as α-(1→2), α-(1→3), α-(1→4) linkages. Dextran can be synthesized from sucrose by different bacterial strains, particularly, species of *Leuconostoc* and *Streptococcus* strains [10]. Dextran has been used extensively in the medical field as a blood plasma volume expander [11] and anti-coagulant [12]. As a macromolecular cross-linker for the CD hydrogel, dextran was oxidized with periodate to produce DA. The aldehyde groups enable the oxidized dextran to react with the free amine groups of SC. The reaction between the aldehyde groups and the amine groups results in the formation of a gel through imine bonds (Schiff base formation) in aqueous solution [13]. This approach eliminates the need for extraneous chemical cross-linkers that may result in undesirable cytotoxic side effects.

A highly oxidized form of CD hydrogel (CD-100) containing 80% oxidized DA (DA-100) has been extensively tested *in vivo* and *in vitro*. CD-100 hydrogel significantly reduced the number of adhesions in both animal and human trials, as well as displaying excellent hemostatic, mucoadhesive, and antimicrobial properties [9,14,15,16]. However, CD-100 hydrogel showed moderate *in vitro* cytotoxicity to Vero cells [2], human nasopharyngeal epithelial cells, and human dermal fibroblasts [17]. DA-100 was found to be the cytotoxic component. While this degree of biocompatibility is tolerated in the setting of ESS [15], it may compromise the use of CD-100 hydrogel as an anti-adhesive in abdominal surgery. Abdominal adhesions pose a significant clinical problem and financial burden to the health sector [18]. Adhesions occur at the site of trauma with a 54% incidence rate after abdominal surgery and a 66% incidence rate after gastrointestinal surgery [19]. The predominant pathogens in post-operative intra-abdominal infections are enteric Gram-negative bacilli, Gram-positive cocci and anaerobic microorganisms, usually sourced from the patient’s endogenous microflora [20,21].

In the hope of extending the application of CD hydrogel for use in abdominal surgery, a reformulation of the hydrogel using a lower oxidized DA (DA-25) was pursued. The hydrogel containing DA-25 (CD-25) was found to be non-cytotoxic to mouse fibroblasts and did not elicit a pro-inflammatory response *in vivo* in BALB/c mice [2]. It significantly reduced the number of adhesions in an *in vivo* porcine hemicolectomy model [22] and was shown to be an effective, nontoxic hemostatic agent to control bleeding in a sheep neurosurgical model [23]. However, before proceeding to human trials, biocompatibility for human dermal fibroblasts (HDFa), and antimicrobial efficacy for bacterial species commonly associated with abdominal infections needed to be determined.

The aim of the present study was to compare the antimicrobial efficacy, *in vitro* biocompatibility, and wound healing capacity of the highly oxidized CD-100 hydrogel with the CD-25, which has a reduced oxidation level. The development of an antimicrobial, biocompatible post-operative hydrogel would provide immense health benefits by reducing the number of adhesions and surgical site infections, particularly in abdominal surgery.

## 2. Results

The CD hydrogel was synthesized according to previously established methods and the details of synthesis are provided elsewhere [2].

### 2.1. Minimum Inhibitory and Minimum Bactericidal Determinations

The antimicrobial activities of CD-100 hydrogel, CD-25 hydrogel, DA-100 and DA-25 were determined against a variety of organisms (Table 1) that are prevalent in post-operative infections by the broth microdilution method. The minimum inhibitory (MIC) and minimum bactericidal concentration (MBC) values for CD-25 hydrogel and DA-25 are shown in Table 2. These compounds were found to be ineffective at inhibiting growth of the aerobic species, *Staphylococcus aureus*, *Escherichia coli*, and *Enterococcus faecalis* at any of the concentrations tested. The anaerobic species, *Peptostreptococcus anaerobius*, *Bacteroides fragilis* and *Clostridium perfringens* were more susceptible to DA-25 than the aerobic bacteria, but CD-25 hydrogel had no detectable activity against anaerobic test species. Of the bacterial strains tested, *Helicobacter pylori* were the most susceptible to DA-25. However, CD-25 hydrogel was ineffective against this organism. Where MIC values were obtained, the MBC was equivalent (Table 2).

**Table 1:** Microorganisms and antibiotic controls used in minimum inhibitory and minimum bactericidal concentration determinations.

Microorganism	American Type Culture Collection Number	Antibiotic Control
*Staphylococcus aureus*	ATCC 9144	Penicillin G
*Escherichia coli*	ATCC 25922	Gentamicin
*Enterococcus faecalis*	ATCC 29212	Gentamicin + Penicillin G
*Bacteroides fragilis*	ATCC 25285	Metronidazole
*Peptostreptococcus anaerobius*	ATCC 27337	Metronidazole
*Clostridium perfringens*	ATCC 13124	Metronidazole
*Helicobacter pylori*	ATCC 11637	Metronidazole

**Table 2:** Minimum inhibitory concentration (MIC) and minimum bactericidal concentration (MBC) of DA-25, CD-25, DA-100, and CD-100 hydrogel against all tested organisms.

Organism	Concentration (mg·mL^−1^)								Positive Controls ^a^ (μg·mL^−1^)	
	DA-25		CD-25		DA-100		CD-100			
	MIC	MBC	MIC	MBC	MIC	MBC	MIC	MBC	MIC	MBC
*Escherichia coli*	>64	>64	>50	>50	32	50	>50	>50	8	8
*Staphylococcus aureus*	>64	>64	>50	>50	8	32	40	40	0.0625	1
*Enterocococcus faecalis*	>64	>64	>50	>50	8	25	>50	>50	0.5	1
*Peptostreptococcus anaerobius*	50	50	>50	>50	4	8	40	40	0.5	1
*Bacteroides fragilis*	50	50	>50	>50	4	8	20	40	0.5	1
*Clostridium perfringens*	32	32	>50	>50	2	2	20	20	1	1
*Helicobacter pylori*	8	8	>50	>50	2	2	20	20	1	1

^a^ Antibiotic control used for each microorganism is shown in Table 1; MIC means minimum inhibitory; MBC means minimum bactericidal concentration.

DA-100 and CD-100 hydrogel were shown to be more effective than CD-25 hydrogel and DA-25, as indicated by lower MIC and MBC values with all the tested organisms (Table 1). Of the aerobic species, *Enterococcus faecalis* was the most susceptible to DA-100 but paradoxically was one of the least susceptible to CD-100 hydrogel. Anaerobic species were more susceptible to DA-100 and CD-100 hydrogel than the aerobic species. CD-100 hydrogel was found to exert bactericidal activity against the anaerobes at the surgical concentration (40 mg·mL^−1^) used in ESS surgery. CD-100 was found to exert an inhibitory effect on *B. fragilis* and *C. perfringens* at 20 mg·mL^−1^ (25 mg·mL^−1^ SC + 15 mg·mL^−1^ DA-100), but not at 25 mg·mL^−1^ (40 mg·mL^−1^ SC + 10 mg·mL^−1^ DA-100). The results of an experiment to determine the effect of anaerobic conditions and culture media on the MIC/MBC indicated no change in values for *E. coli* 25922 compared to standard aerobic incubation (data not shown).

### 2.2. Antimicrobial Mode of Action

**Figure 1:**
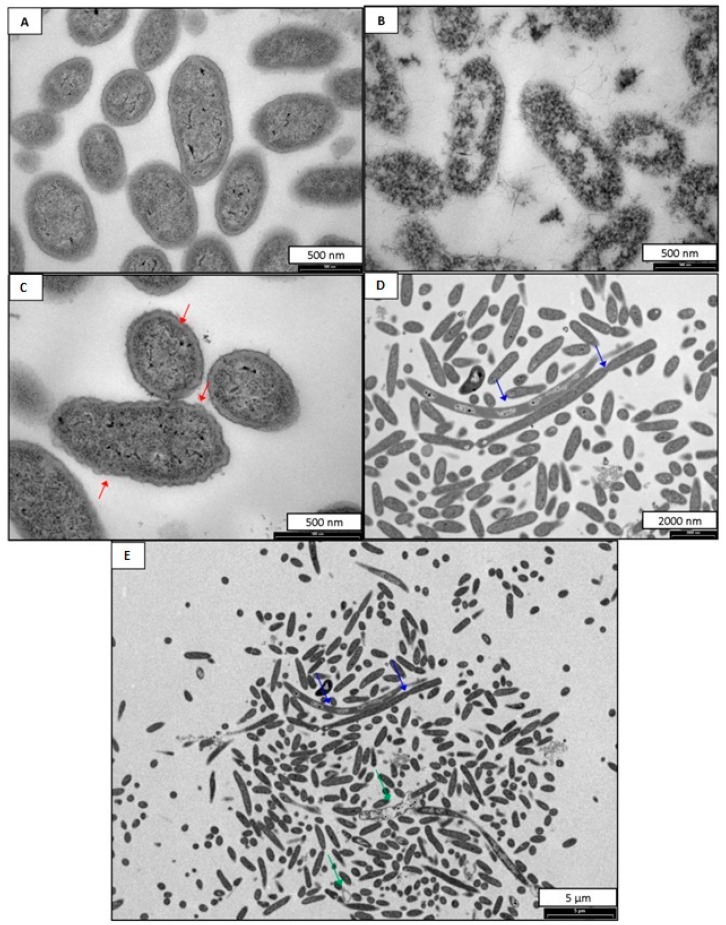
TEM images of *B. Fragilis* (**A**) *B. Fragilis* untreated; (**B**) *B. Fragilis* treated with 0.02% Triton X-100; (**C**–**E**) *B. Fragilis* treated with 4 mg·mL^−1^ of DA-100. Red arrows indicate cell membrane rippling. Blue arrows indicate areas of incomplete cell division. Green arrows indicate bacterial ghosts.

**Figure 2:**
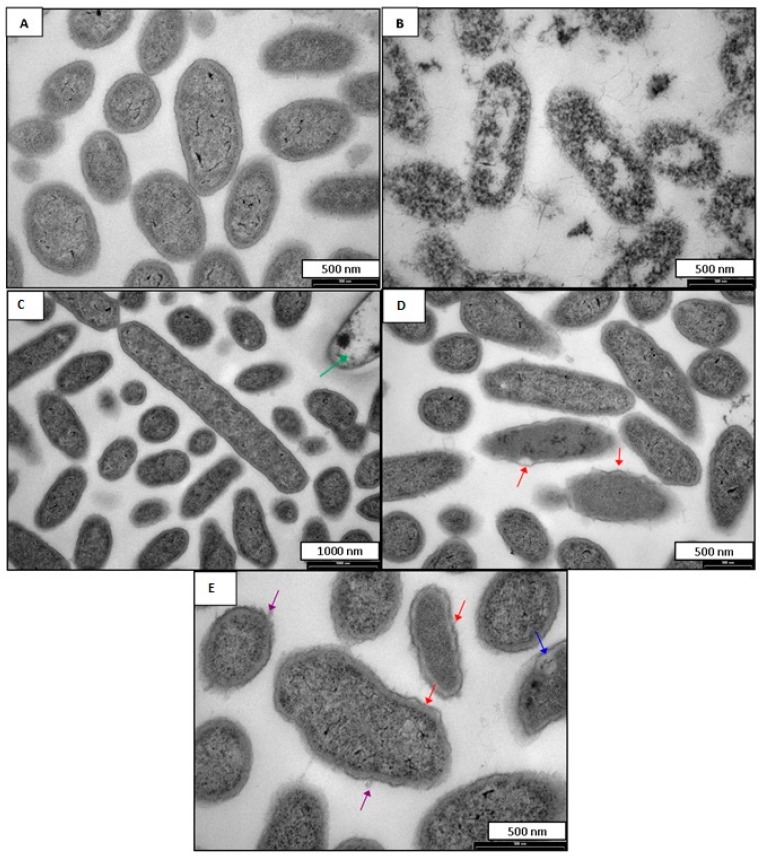
TEM images of *B. fragilis* (**A**) *B. fragilis* untreated; (**B**) *B. fragilis* treated with 0.02% Triton X-100; (**C**–**E**) *B. fragilis* treated with 20 mg·mL^−1^ of CD-100. Red arrows indicate cell membrane rippling and separation of the cell wall from cytoplasmic contents. Purple arrows indicate blebbing. Green arrows indicate bacterial ghosts. Blue arrows indicate an area of reduced electron density.

The susceptibility of the anaerobes studied to DA-100 and CD-100 prompted an evaluation of the cellular effects using transmission electron microscopy (TEM). TEM images for *B. fragilis* incubated with DA-100 and CD-100 hydrogel are shown in Figure 1 and Figure 2 respectively. Untreated *B. fragilis* cells appeared round with clear and discrete membranes (Figure 1A and Figure 2A). *B. fragilis* cells incubated with 0.02% Triton X-100 served as a positive control (Figure 1B and Figure 2B). As expected, these cells completely lost their cell wall integrity and were lysed. Bacterial cells treated with DA-100 at 4 mg·mL^−1^ showed disruption of the cell membrane characterized by cell wall rippling and blebbing (Figure 1C). Another notable observation seen in cells treated with DA-100 was the appearance of greatly enlarged and elongated cells measuring at ~9 nm in length, suggesting the *B. fragilis* cells were incapable of completing cell division (Figure 1D). Treatment with DA-100 also gave rise to the appearance of bacterial ghosts (Figure 1E). A similar effect was observed with cells incubated with 20 mg·mL^−1^ of CD-100 hydrogel (Figure 2). Bacterial cells showed signs of cell membrane disruption, cell wall rippling, and blebbing, in addition to the cell wall separating from the cell membrane (Figure 2D,E). As observed in cells treated with DA-100, bacterial ghosts were seen with treatment with CD-100 hydrogel (Figure 2C).

### 2.3. Cytotoxicity Assay

**Figure 3:**
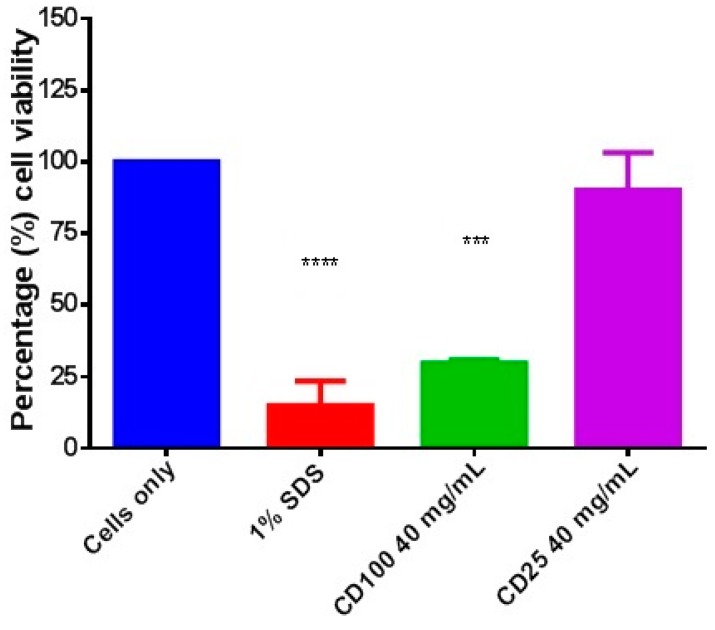
*In vitro* cytotoxicity evaluations of CD-100 and CD-25 hydrogel at 40 mg·mL^−1^. Cells only serve as a negative control and 1% sodium dodecyl sulfate (SDS) served as the cytotoxic positive control. Data are presented as mean ± SD performed using one-way analysis of variance (ANOVA) followed by a Bonferroni’s multiple comparison. (*** *p* < 0.001), (**** *p* < 0.0001) as compared to cells only control.

Cytotoxicity of the hydrogels was evaluated by incubating CD-25 and CD-100 in Transwell inserts with HDFa cells for 48 h and assessing cell viability by the MTT assay. Treatment with 40 mg·mL^−1^ of CD-100 hydrogel significantly reduced cell viability to 30% (*p* < 0.001) compared to the cells only control (Figure 3). Cells treated with 40 mg·mL^−1^ of CD-25 displayed a minor reduction (10%) in cell viability. However this difference was not statistically significant when compared to the cells only control.

### 2.4. In Vitro Wound Healing Assay

**Figure 4:**
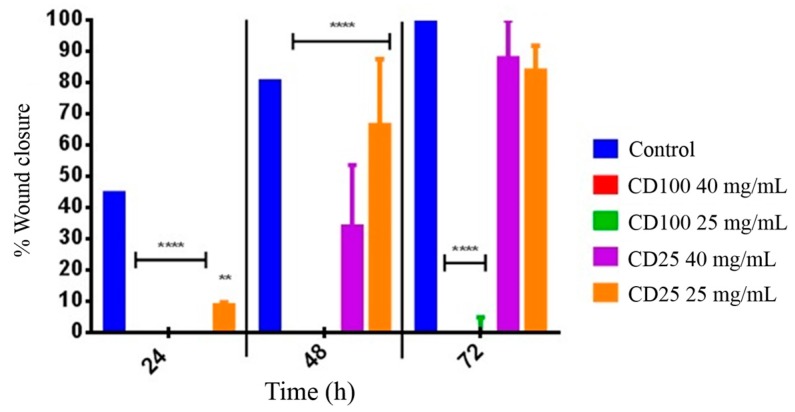
Wound healing assay displaying percentage wound closure of dermal fibroblast cells in an IBIDI insert cell culturing system at 24, 48, and 72 h. HDFa cells were treated with (red) 40 mg·mL^−1^ CD-100, (green) 25 mg·mL^−1^ CD-100, (purple) 40 mg·mL^−1^ CD-25 and (orange) 25 mg·mL^−1^ CD-25. Untreated cells served as a control. Data are presented at mean ± SD statistically significant groups were determined by 2-way ANOVA followed by a Bonferroni multiple comparisons test. (** *p* < 0.01), (**** *p* < 0.0001) as compared to control.

Decreasing wound size is an important aim in the mechanism for wound healing. Cytotoxic material would be expected to inhibit cell migration. Images of the human dermal fibroblast (HDFa) cells at varying incubation times (0, 24, 48, 72 h) showed the migration of the fibroblast cells across the 500 μm gap created by the IBIDI inserts containing either CD-100 hydrogel, CD-25 hydrogel, or cells only (negative control) (Figures S1 and S2). Percentages of wound closure for each treatment are shown in Figure 4. In the cells only control, fibroblast cells appear to begin migrating at 24 h, resulting in approximately 45% wound closure at 24 h, 80% wound closure at 48 h, and complete closure at 72 h (Figures S1A and S2A). For cells incubated with CD-100 hydrogel at both 40 and 25 mg·mL^−1^, there is no observed fibroblast cell migration at any time point (Figure S1) as well as no wound closure (Figure 4). The differences between the CD-100 hydrogel results and the cells only control were statistically significant at all time-points tested (*p* < 0.0001). Cells incubated with 25 mg·mL^−1^ CD-25 hydrogel showed some reduction in the speed of cell migration and wound closure (Figure S2). Wound closure was 10% at 24 h, 65% closure at 48 h and 85% closure at 72 h (Figure 4). With treatment of 40 mg·mL^−1^ of CD-25 hydrogel, HDFa cells also showed impaired cell migration when compared to the cells only control (Figure S2). There was no observed cell migration at 24 h (*p* < 0.0001), 35% wound closure at 48 h (*p* < 0.0001), and approximately 90%–100% wound closure at 72 h (Figure 4). However, for both concentrations of CD-25 hydrogel, the percentage closure at 72 h was not statistically significantly different from the cells only control.

## 3. Discussion

Hydrogel application during abdominal surgery can significantly reduce the number of adhesions by acting as an effective intraperitoneal barrier [24]. An antimicrobial hydrogel would provide the added benefit of significantly decreasing infection-related complications. The ideal surgical hydrogel must be biocompatible, biodegradable, and facilitate the healing process. Viscous solutions of high-molecular-weight polymers, such as solutions of carboxymethylcellulose (CMC), hyaluronate, icodextrin, poly-l-lysine, and polyethylene glycol (PEG) have been studied, but have shown mixed efficacy results [25]. The CD hydrogel developed for ESS (CD-100 hydrogel) has been shown to be an effective adhesion prevention adjunct [26] in addition to displaying significant antimicrobial activity [9]. Another group has recently reported on the antibacterial properties of dextran-based hydrogels as bioadhesives, and found them to be biocompatible in the presence of human erythrocytes [27]. However, our CD-100 hydrogel was found to be moderately cytotoxic in *in vitro* assays, with DA-100 identified as the cytotoxic component [17]. In order to broaden the application of the CD hydrogel for abdominal surgery application, a lower oxidized version of the DA (DA-25) and its corresponding hydrogel (CD-25) were investigated for antimicrobial efficacy and biocompatibility in comparison to the more highly oxidized forms (DA-100 and CD-100 hydrogel).

MIC and MBC determinations showed that the highest tested concentrations of DA-25 (64 mg·mL^−1^) and CD-25 hydrogel (50 mg·mL^−1^) were less effective than DA-100 and CD-100 hydrogel at the same concentration with all the bacterial species tested. The difference in antimicrobial efficacy between formulations of DA is thought to be due to differences in oxidation levels. DA-100 is 80% oxidized, and consequently possesses a greater number of reactive aldehyde groups than DA-25, which is 25% oxidized. The reactive aldehyde groups of DA are thought to be responsible for the antimicrobial activity of CD hydrogel [9,17]. This activity is considered to be similar to that observed for other aldehyde containing biocides, such as glutaraldehyde and orthophthaldehyde, where the aldehyde groups are reported to react with primary amine groups present on bacterial cell walls, resulting in a strong adhesive effect [28,29]. CD-25 hydrogel was found to be ineffective at inhibiting and killing, any of the microbes at any of the proposed surgical concentrations (40 and 25 mg·mL^−1^). The anaerobic organisms, *B. fragilis*, *P. anaerobius* and *C. perfringens*, as well as the microaerophilic *H. pylori* were more susceptible than the facultative anaerobic microbes *E. coli*, *S. aureus* and *E. faecalis*, being generally inhibited with much lower concentrations of DA-100 and CD-100 hydrogel. These facultative anaerobes can metabolize the oxygen present in dextran aldehyde that would typically be toxic to obligate anaerobes. Since the majority of dermal wounds and intra-abdominal infections are likely to be colonized by a polymicrobial population, in hypoxic tissue the proliferation of facultative aerobes would consume any residual oxygen and thereby promote the growth of fastidious anaerobes, such as the *Bacteroides* species [30]. Although bactericidal efficacy of DA-25 and CD-25 is decreased, the use of prophylactic antibiotics at the time of surgery would help reduce the number of surgical site infections, allowing protection against some but not all microbes as antimicrobial resistance is common [31].

The susceptibility of the anaerobes to DA-100 and CD-100 hydrogel prompted an evaluation of the cellular effects on *B. fragilis* using TEM. As previously reported for DA-100 and CD-100 hydrogel, the primary site of action is believed to be the bacterial cell membrane. For *B. fragilis* incubated with DA-100 or CD-100 hydrogel, blebbing was seen at the cell wall. In eukaryotic cells cytoplasmic blebbing is indicative of apoptosis, or necrosis [32,33]. Other studies have shown the manifestation of blebbing at the cell surface to be indicative of bacterial lysis [34,35,36]. A notable observation in *B. fragilis* cells incubated with DA-100 was the appearance of greatly enlarged and elongated rods. DA-100 may target and bind to proteins responsible for cell division with disruption resulting in elongated cells.

As anticipated, the CD-100 hydrogel was found to be moderately cytotoxic for human dermal fibroblasts. CD-25 demonstrated considerably less cytotoxicity than CD-100, as no changes in cell morphology were observed and there was no statistically significant reduction in cell viability compared to the solvent (culture medium only) control as assessed by the MTT assay. This is consistent with observed *in vitro* cytotoxicity assay results for CD-100 hydrogel previously obtained using the xCELLigence system (Roche Applied Bioscience, Mannheim, Germany) in Vero cells [2], human nasopharyngeal epithelial cells, and dermal fibroblasts [17].

Wound healing assays performed with human dermal fibroblasts reflected the cytotoxicity assay results. CD-100 inhibited the proliferation and migration of fibroblasts resulting in incomplete wound closure after 72 h. The anti-proliferative effect of the CD-100 hydrogel is thought to prevent adhesion formation by simply limiting fibrin deposition. In contrast to the CD-100 hydrogel, a much lower level of inhibition of cell migration was seen with CD-25 and, by the end of the experiment (72 h), migration was comparable to the cells only control. Thus, CD-25 hydrogel displayed markedly improved biocompatibility and wound healing *in vitro* compared to the more highly oxidized counterpart, CD-100 hydrogel.

## 4. Materials and Methods

### 4.1. Materials

All culture media were Becton Dickson (BD) sourced from Fort Richard Laboratories (Auckland, New Zealand) unless otherwise stated. Media were prepared according to the manufacturer’s instructions. Supplemented Brucella broth (BB) was prepared with the addition of 1% hemin and vitamin K, and sterilized for 15 min at 121 °C as stated in the Clinical and Laboratory Standards Institute (CLSI) performance standards [37]. BB supplemented with fetal calf serum (FCS) (Invitrogen™, Life Technologies, Auckland, New Zealand) was prepared with filter-sterilized serum added after the autoclaved medium had cooled to room temperature. Bacterial strains were obtained from New Zealand Reference Collection (Environmental Science and Research, Porirua, New Zealand). Antibiotics were from Sigma-Aldrich (Castle Hill, New South Wales, Australia) except for gentamicin, which was purchased as a sterile solution from Life Technologies (Auckland, New Zealand). Human adult dermal fibroblast cell line (HDFa) was purchased from Biologics™ Company (Portland, OR, USA). The following were supplied by Life Technologies (Auckland, New Zealand): Medium 106 supplemented with 1% low serum growth supplement,); Tryp-LE™ Express Enzyme (Gibco, Carlsbad, CA, USA; tissue culture flasks and plates (BD Falcon™, Heidelberg, Germany; and transwell inserts (8.0 μm pore size; BD). Phosphate buffer chemicals were purchased from Scharlau Chemie (Barcelona, Spain).

### 4.2. Bacterial Strains

The microorganisms used in this present study (Table 1) included: *E. faecalis*, *S. aureus* and *E. coli*, which were maintained on tryptic soy agar (TSA) and in Mueller Hinton cation adjusted media (MH) for MIC and MBC testing. *P. anaerobius*, *B. fragilis* and *C. perfringens* were maintained on supplemented Brucella agar (1% vitamin K and 1% hemin) in anaerobic conditions. *H. pylori* cultures were maintained on 5% sheep blood agar incubated at 37 °C in microaerophilic conditions.

### 4.3. Preparation of DA, SC, and CD Hydrogel

The 80% oxidized dextran aldehyde (DA-100) (Batch no. NZP 9911103) and the 25% oxidized dextran aldehyde (DA-25) (Batch no. NZP P2011303) and SC were prepared as previously described [17]. Briefly, SC and DA were dissolved in 0.24% sodium phosphate buffer, 7.4 pH (NaPB was prepared by mixing 13.5 mmol·L^−1^ disodium hydrogen phosphate with 4 mmol·L^−1^ sodium dihydrogen phosphate). The DA solutions were filter sterilized using 0.22 μm membrane filters (Merck Millipore, Manukau, New Zealand). SC (Batch no. NZP9911110) was prepared at a concentration of 100 mg·mL^−1^ by dissolving in NaPB. SC was sterilized by autoclaving at 121 °C. The CD hydrogels were formed using a 1:1 mixture of SC and DA with the concentrations described in Table 3.

### 4.4. Antimicrobial Efficacy Testing

The MICs and MBCs of DA-100, DA-25, CD-100, and CD-25 were determined by the broth micro-dilution method adapted from the CLSI performance guidelines [37]. All other conditions (incubation times and conditions and media supplements) were as specified in CLSI guidelines for aerobic and anaerobic bacteria. The range of concentrations tested is listed in Table 4 along with the antibiotic controls. Briefly, inocula were prepared by adjusting overnight cultures to 10^6^ CFU·mL^−1^ in the appropriate double strength culture medium, which were then referenced to standard curves of optical density versus viable count. Optical density readings were obtained from a spectrophotometer (Novaspec II visible, Cambridge, UK). Equal volumes (100 μL) of inocula and dilutions of DA, SC, CD hydrogel and antimicrobial controls prepared in NaPB were mixed in 96-well microtiter plates (Falcon, BD company) to give a final concentration of 5 × 10^5^ CFU·mL^−1^ followed by incubation at 37 ± 2 °C for 24 h in the appropriate atmospheric condition. Wells were examined for growth to determine MIC. For MBC, wells with no apparent growth were subcultured on to the appropriate agar plates and examined for growth after 24 h incubation at 37 ± 2 °C. MIC and MBC determinations where carried out in triplicate in two independent experiments.

**Table 3:** Table of the concentrations used in the formation of the CD hydrogel. CD hydrogel final concentration is calculated to reflect a dilution, as the hydrogel is formed using a 1:1 mixture of the concentration of SC and DA.

CD hydrogel Final Concentration (mg·mL^−1^)	Concentration of CD Hydrogel Components (mg·mL^−1^)	
	Succinyl Chitosan (SC)	Dextran Aldehyde (DA)
50	50	50
40 ^a^	50	30
25 ^b^	40	10
20	25	15
10	12.5	7.5
5	6.25	3.75
2.5	3.125	1.875
1.25	1.56	0.94

^a^ denotes the proposed surgical concentration utilized for ENT surgery; and ^b^ denotes the surgical concentration proposed for *in vivo* use for abdominal surgery.

To investigate the effect of anaerobic incubation conditions on MIC and MBC values, broth micro-dilutions were performed with DA-25 and DA-100 under anaerobic conditions using the facultative anaerobe *E. coli* 25922. Media prepared for all anaerobic work was all pre-reduced in an anaerobic chamber 24 h prior to experimentation. In order to assess the effect of culture media, the assays were performed in parallel with MH broth and supplemented BB in triplicate in two independent experiments.

**Table 4:** Concentrations of compounds used in MIC and MBC determinations.

Compounds	Concentration (mg·mL^−1^)
DA-100	64, 50, 32, 16, 8, 4, 2
DA-25	64, 50, 32, 16, 8, 4, 2
CD-100	50, 40, 25, 20, 10, 5, 2.5, 1.25
CD-25	50, 40, 25, 20, 10, 5, 2.5, 1.25
^a^ Penicillin G	0.032, 0.016, 0.008, 0.004, 0.002, 0.001, 0.0005, 0.00025, 0.000125
^a^ Gentamicin	0.032, 0.016, 0.008, 0.004, 0.002, 0.001, 0.0005, 0.00025
^a^ Metronidazole	0.016, 0.008, 0.004, 0.002, 0.001, 0.0005, 0.00025, 0.000125
^a^ Penicillin G + Gentamicin	0.032/0.032, 0.016/0.016, 0.008/0.008, 0.004/0.004, 0.002/0.002, 0.001/0.001, 0.0005/0.0005

^a^ indicates antimicrobial controls.

### 4.5. Transmission Electron Microscopy (TEM)

*B. fragilis* was prepared for TEM as previously described [38]. Overnight cultures of *B. fragilis* grown anaerobically in supplemented BB were added in 1 mL volumes to 500 μL of either CD-100 hydrogel or DA-100. NaPB (0.24%) and Triton X-100 (0.02%) served as the negative and positive controls, respectively. The concentrations of CD-100 and DA-100 were determined from the MIC values obtained for *B. fragilis*; these were 20 mg·mL^−1^ (2.5% SC + 1.5% DA-100) of CD-100 hydrogel and 4 mg·mL^−1^ of DA-100. After incubation for 4 h at 35 ± 2 °C in an anaerobic chamber, the cells were washed twice with 5 mmol·L^−1^ sodium phosphate buffered saline (NaPB pH 7.2). The supernatant was removed and replaced with 2.5% glutaraldehyde in 0.08 M phosphate buffer and fixed on a rotator at room temperature for 2 h. The cells were washed three times in 0.08 M PB and fixed in 1% osmium tetroxide in 0.08 M PB buffer overnight at 4 °C. Following dehydration in an ethanol gradient, cells were embedded in Spur’s epoxy resin. Thin sections of the specimens were cut with a diamond knife on an Ultracut Ultramicrotome (Reichert, Depew, NY, USA). The grids were examined with a Philips CM100 BioTWIN transmission electron microscope (Philips/FEI Corp, Eindhoven, Holland), LaB6 emitter fitted with MegaView III digital camera (Olympus Soft Imaging Solutions GmbH, Münster, Germany).

### 4.6. Cytotoxicity Assay

CD hydrogel cytotoxicity was assessed by an MTT assay following the ISO standard 10993-05 guidelines for indirect testing of a medical device. *In vitro* cell toxicity was carried in 24-well plates (Falcon, BD falcon) at a seeding density of 0.5 × 10^5^ cells·mL^−1^. The hydrogels were prepared in Transwell cell culture inserts by mixing 100 μL of SC (50 mg·mL^−1^) with 100 μL of DA (30 mg·mL^−1^). Untreated cells served as a growth control. When the hydrogel had fully gelled, the inserts were transferred into the 24-well tissue culture plates and incubated for 48 h. For a positive cytotoxic cell control, 100 μL of 10% SDS were added to the insert, and equivalent amount of media was added to serve as an untreated control. At the end of the incubation period, the Transwell inserts were removed and the cell morphology observed by light microscopy. Cell viability was then assessed by an MTT assay [39]. The absorbance at OD_570nm_ (650_nm_ reference) was measured with a plate reader (Varioskan Flash, Thermo Fisher Scientific, Vantaa, Finland). Mean OD values were calculated from triplicate replicates in duplicate experiments.

### 4.7. In Vitro Wound Healing Assay

This experiment was carried out using petri μ-dishes (IBIDI GmbH, Am Klopferspitz Martinsried, Munich, Germany) with inserts that allow cells to grow in two designated areas (each 0.22 cm^2^) with a predetermined gap size of 500 ± 50 μm. To each well, 70 μL of human dermal fibroblast cells were seeded at a concentration of 3 × 10^5^ cells·mL^−1^ and incubated at 35 ± 2 °C in a humidified 5% CO_2_ atmosphere for 24 h or until the cells were 70%–80% confluent. After removal of the insert, the cell free gap was filled with 10 μL of CD-100 or CD-25 hydrogel. The concentrations of the CD hydrogels tested were 40 and 25 mg·mL^−1^. Cells alone without test samples served as a negative control. The cells were subsequently covered with 2 mL of supplemented Medium 106 and incubated at 35 ± 2 °C in a humidified 5% CO_2_ atmosphere for 72 h. The proliferation and migration of the cells over the cell free gap was investigated by image capture at designated time-points (0, 24, 48 and 72 h) using an inverted microscope (Olympus IX70 Ltd., Osaka, Japan) at ×10 magnification. The digitalized images were analyzed by ImageJ software (Rasband, W.S., ImageJ, U.S. National Institute of Health, Bethesda, Maryland, USA, http://rsb.info.nih.gov/ij/, 1997–2014). The percentage wound closure at the different time-points was calculated by the equation below. At least three different images were analyzed for each sample and for each time point.

$$\text{Wound closure \% = }\left(1-Wound\;area\;at\;T_{\text{t}}÷Wound\;area\;at\;T_{0}\right)\;\times \;100\%$$

*T*_t_ is the time afer wounding. T_0_ is the time immediately after wounding. The results were presented as mean ± SD for two independent experiments.

### 4.8. Statistical Analysis

Statistical analysis was performed using two-way ANOVA followed by Bonferroni’s multiple comparisons test. Significant differences were set at * *p* < 0.05, ** *p* < 0.01, *** *p* < 0.001, and **** *p* < 0.0001.

## 5. Conclusions

The *in vitro* experiments performed in this study demonstrate the importance of DA oxidation level in the biocompatibility and antimicrobial efficacy of a chitosan, dextran based surgical hydrogel. Our results indicate that DA-25, although more biocompatible that DA-100, has reduced antimicrobial ability and anti-fibroblast proliferative effect. Despite this, the CD-25 hydrogel still proved effective in significantly reducing the number of adhesions in a porcine abdominal surgery model [22] and in human ESS trials [40]. It is thought that the hydrogel functions as an effective physical adhesion barrier, working by keeping traumatized, opposing surfaces apart long enough to avoid tissue adherence [41,42]. The CD-25 hydrogel biodegrades in a timely manner as shown by *in vivo* studies using BALB/c mice. The CD-25 hydrogel was found to biodegrade in under a week after subcutaneous injection and under three days after intraperitoneal injection [43].

Future studies incorporating broad-spectrum antimicrobial agents into the hydrogel could be pursued to increase the functionality of the gel. In addition, a DA with a more moderate level of oxidation (~40%–60%) could result in a CD hydrogel that combines the benefits of biocompatibility, antimicrobial efficacy, and adhesion prevention. The development of a surgical hydrogel that is hemostatic, antimicrobial, and prevents adhesion formation would be of immense benefit leading to reduced healing times, lowered infection rates, and eliminate the need for second look surgeries to remove adhesions.

## Supplementary Materials

Migration of dermal fibroblast cells co-incubated with CD-100 (Figure S1) or CD-25 (Figure S2) hydrogel using IBIDI cell culture insert system. Images were captured at time 0, 24, 48, and 72 h incubation time using an OlympusIX70 microscope. Supplementary materials can be found at http://www.mdpi.com/1422-0067/16/06/13798/s1.
