# Supplementary material for: Reducing the Oxidation Level of Dextran Aldehyde in a Chitosan/Dextran-Based Surgical Hydrogel Increases Biocompatibility and Decreases Antimicrobial Efficacy
Source: Int J Mol Sci. 2015 Jun 16;16(6):13798–814. doi: 10.3390/ijms160613798 (PMC4490524; doi:10.3390/ijms160613798)
Supplement: Supplementary file 1 [file ijms-16-13798-s001.pdf]

Supplementary Information

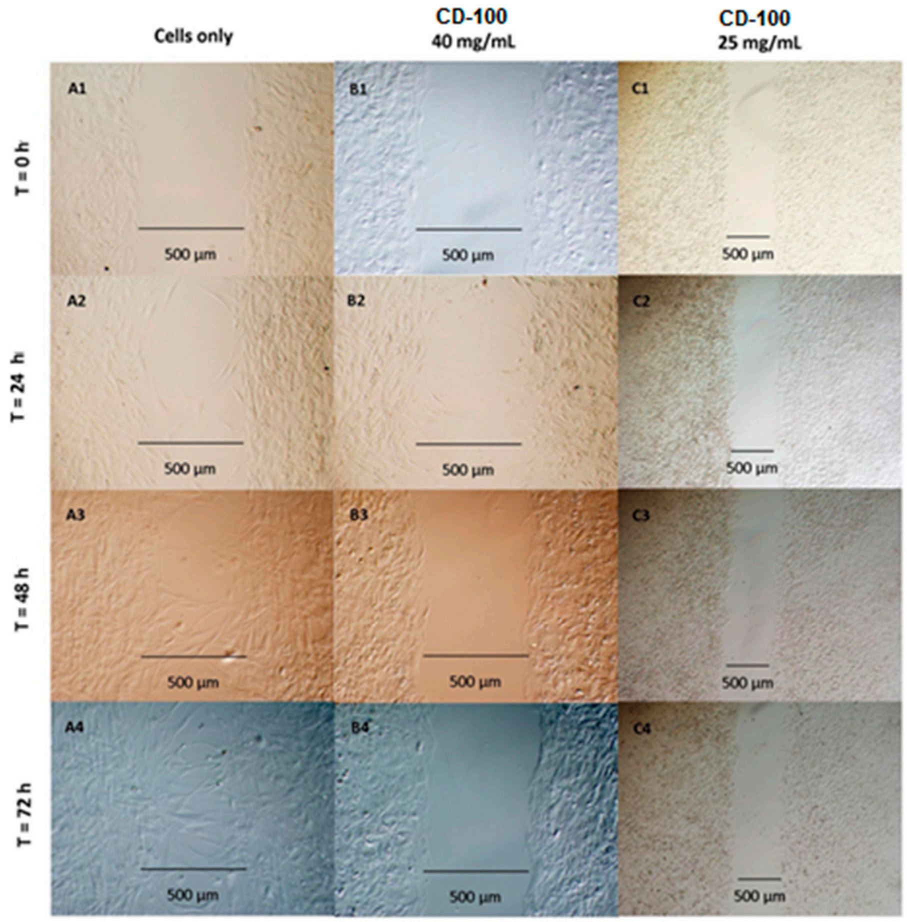

(**A**) (**B**) (**C**)

Figure S1. Migration of dermal fibroblast cells only (**A**); fibroblast cells co-incubated with (**B**) 40 mg·mL^{-1} and (**C**) 25 mg·mL^{-1} of CD-100 using an IBIDI cell culture insert system. Images were captured at time 0, 24, 48, and 72 h incubation time using an Olympus IX70 microscope.

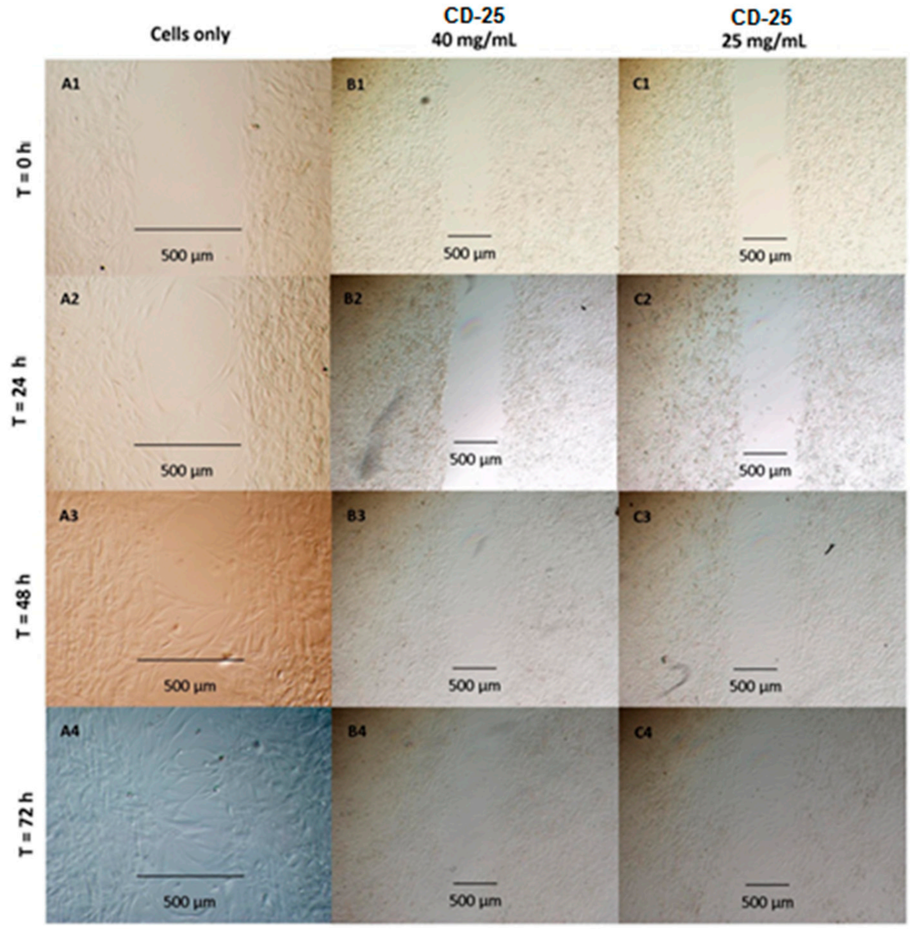

(**A**) (**B**) (**C**)

Figure S2. Migration of dermal fibroblast cells only (**A**); fibroblast cells co-incubated with (**B**) 40 mg·mL^{-1} and (**C**) 25 mg·mL^{-1} of CD-25 using an IBIDI cell culture insert system. Images were captured at time 0, 24, 48, and 72 h incubation time using an Olympus IX70 microscope.
